# A Case of Buccal Abscess from an Impacted Wisdom Tooth in an Elderly Person with Malnutrition

**DOI:** 10.1155/2016/9437514

**Published:** 2016-11-09

**Authors:** Yuki Kojima, Mizuha Kojima, Kazuhiro Sakaguchi, Yutaka Sakaguchi

**Affiliations:** ^1^Department of Physiology, Tokyo Dental College, 2-9-18 Misaki-cho, Tokyo 101-0061, Japan; ^2^Sakaguchi Dental Clinic, 1-20-5 Kasuga, Chuo-ku, Chiba 260-0033, Japan

## Abstract

We report a case of buccal abscess caused by an impacted wisdom tooth in an extremely elderly person with malnutrition. The patient was a 94-year-old man, who complained that he had found it hard to open his mouth and that his cheek had been swollen for the previous 2 weeks. He had a shallow oral wound caused by an improperly fitting denture; however, the wound became infected. We performed incisional drainage of the abscess under local anesthesia. The swelling disappeared and he was able to open his mouth 55 mm. The elderly have a high risk of healing failure of injuries and it has been reported that infection in a host in a compromised state is severely intractable. This elderly patient was in a compromised state because of malnutrition. Cases such as this one will increase as the elderly population increases. Dentists need to consider the quality of life of patients with a longer life expectancy and should offer patients several treatment options before their general condition deteriorates.

## 1. Introduction

In recent years, the number of cases of elderly individuals with a deep abscess in the maxillofacial area because of tooth infection has increased [1]. Abscesses in these cases are sometimes related to impacted teeth and nonvital teeth [2–4]. Although it is not uncommon that dentures can cause stomatitis or even wounds, this is less likely to result in the formation of an abscess. In this report, we report a case of buccal abscess derived from an impacted wisdom tooth in a person of marked elderly age.

## 2. Case Presentation

A 94-year-old man felt discomfort in the region of his right mandibular molars for the previous month. He complained that he found it hard to open his mouth and that his cheek had been swollen for the previous 2 weeks. He had difficulty eating but could not gain admission to hospital. When his cheek became painful, he requested a house-call from the dental clinic through his family. He had a history of atrial fibrillation and hypoalbuminemia. He commonly used clopidogrel sulfate (Plavix®, Sanofi, Paris, France) and a protein amino acid formulation (Ensure liquid, Meiji, Tokyo, Japan).

**Figure 1:**
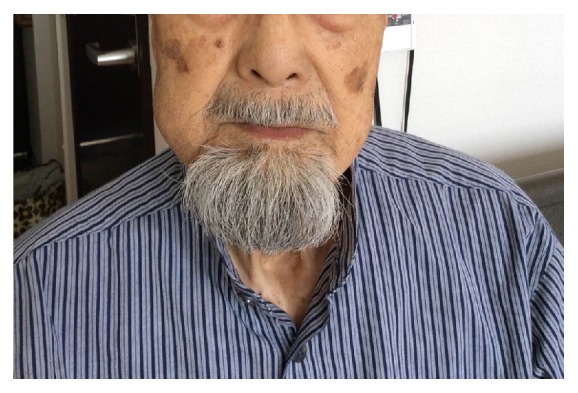
Swelling was seen in the right cheek.

**Figure 2:**
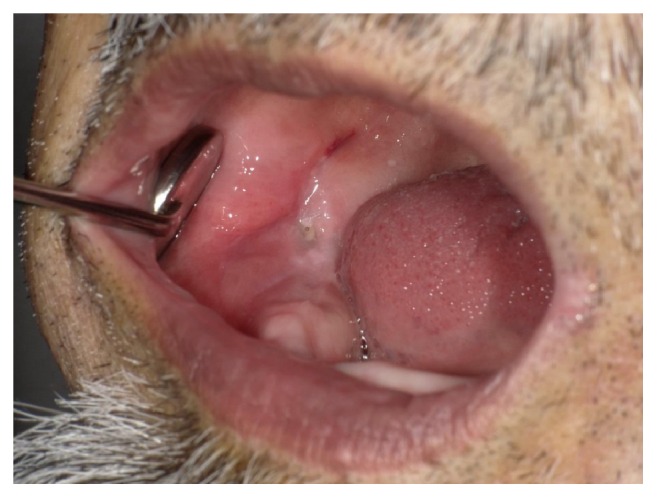
An inner tooth fistula could be observed in the lower right corner. The fistula exuded pus from the impacted wisdom tooth.

At the initial visit, his right cheek and submandibular region were swollen and showed mild reddening (Figure 1). He felt tenderness in the right submandibular lymph nodes. His mouth-opening distance was 23 mm (between the residual ridges). He had no fever (36.4°C) and no pain on swallowing. There was swelling and a wave of redness in the buccal mucosa and the region of the alveolar ridge corresponding to the molars. We observed a fistula inside the mouth in the region of the right molars (Figure 2). This fistula discharged a foul-smelling, yellowish-white pus.

**Figure 3:**
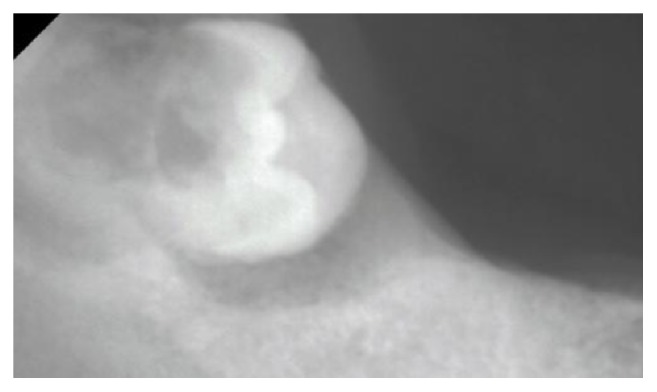
A bone transmission image was seen around the tooth crown.

He reported that all his teeth had already been extracted; however, a right impacted wisdom tooth was noted by palpation. We then inspected the impacted wisdom tooth using a portable X-ray imaging apparatus (Dexco ADX4000W®, Dexcowin, Pasadena, CA, USA) (Figure 3). A bone transmission image of the tooth was obtained, and there was inflammation around the tooth that suggested impaction of the tooth. The trailing edge of his lower denture was broken and sharp, and he reported that this had irritated his mucosa for 1 month. The wound caused by the denture colocalized with the fistula; we therefore considered that the route of infection was via the wound caused by the ill-fitting denture. We diagnosed a mild buccal abscess at the impacted wisdom tooth.

We explained his condition to him and then performed incisional drainage under local anesthesia. We dissected about 1 cm of the mucosa above the impacted wisdom tooth using a #15 knife. We observed a discharge of pus and placed a gauze drain in position. We prescribed cephem antibiotic 100 mg (3 times per day) for 3 days (Cefcapene®, Towa Pharmaceutical Co., Osaka, Japan). On the first postoperative day (POD), the pain disappeared and drainage stopped. By POD 7, his swelling had disappeared and his mouth-opening distance had extended to 55 mm. We considered tooth extraction of the wisdom teeth as the source of infection, given that the third molar teeth fulfill no purpose. After consultation with the responsible physician, we ascertained that his serum albumin had been 3.4 g/dL a month previously. His chronic malnutrition was related to loss of appetite, as he was not very adept at activities of daily living.

We explained to him that the only treatment option was to remove the impacted tooth and that he had a risk of healing failure after extraction of the tooth due to his malnourished state. He had experienced prolonged pain because of healing failure after tooth extraction 20 years earlier and did not want to undergo any surgical procedure if possible because he was elderly. He thus selected to avoid further surgical intervention. We therefore observed him carefully and performed oral care regularly, and his inflammation did not recur. Although the fistula did not disappear, it became smaller. We continued following this patient up, keeping a future risk of infection in mind.

## 3. Discussion

Injuries of the elderly have a high risk of healing failure [5, 6]. In addition, the patient described here was in a compromised state because of malnutrition. Poor appetite was associated with higher risk of poor healthcare outcomes from the viewpoint of either basic research or clinical research [7, 8].

The wound caused by the ill-fitting denture was not so deep that he complained of pain; however, infection arose from this wound. It has been reported that infection in a host in a compromised state is severely intractable [4, 8]. The buccal space sometimes becomes involved when infection of the maxillary molars occurs superior to the attachment of the buccinator muscles [9]. Infection in this space has a dramatic appearance and may cause trismus. Surgical drainage is crucial for resolving dental abscesses [10, 11].

In this case, we not only administered drug therapy, but also performed surgery for rapid control of infection, which resulted in a good outcome. However, we did not remove the cause of inflammation as we did not extract his impacted wisdom tooth, as this was contrary to the patient's wishes. Thus, there was a future inflammation risk, and the patient requires continued careful observation.

Cases such as this one will increase as the elderly population increases. The patient first received full dentures more than 20 years earlier and had not had his dentures checked for 10 years, as he could not go to the dental clinic on his own. It is likely that the number of patients who have systemic disease and have such traveling difficulty will increase.

When dentists create dentures for seemingly edentulous patients, they sometimes fail to do a thorough examination, such as taking an X-ray image. If an impacted tooth does not hamper making the denture, dentists should explain the future risks involved in not treating the impacted tooth. Furthermore, we propose that dentists should consider the quality of life of patients, particularly individuals who longer lived, and should recommend several treatment options to such patients before their general condition deteriorates.
